# Where and How Breastfeeding Promotion Initiatives Should Focus Its Attention? A Study from Rural Wardha

**DOI:** 10.4103/0970-0218.66865

**Published:** 2010-04

**Authors:** A R Dongre, P R Deshmukh, A P Rawool, B S Garg

**Affiliations:** Department of Community Medicine, Sri Manakula Vinayagar Medical College, Pondicherry, India; 1 Dr. Sushila Nayar School of Public Health, Mahatma Gandhi Institute of Medical Sciences, Sewagram – 442 102, India

**Keywords:** Breastfeeding, health education, IMNCI

## Abstract

**Background:**

In India, the practice of breastfeeding is almost universal, but initiation of breastfeeding is generally quite late and colostrum is discarded. Integrated Management of Neonatal and Childhood Illness (IMNCI) strategy recommended systematic assessment of breastfeeding and emphasized counseling of the mother on proper positioning and attachment of infant to the breast.

**Objective:**

To assess breastfeeding among mothers of below six months children in rural Wardha.

**Materials and Methods:**

The present cross-sectional study was undertaken in surrounding 23 villages of Kasturba Rural Health Training Center (KRHTC), Anji. Two Auxiliary Nurse Midwives (ANMs) trained in IMNCI paid house visits to 99 mothers during the study period and undertook the assessment of breastfeeding using IMNCI assessment form for young infants. Auxiliary Nurse Midwives observed and recorded the positioning and attachment of infant to the breast as per IMNCI guidelines. The data were entered and analyzed using Epi_Info (version 6.04d) software package.

**Results:**

Most of the deliveries 94 (94.9%) took place in the healthcare facilities. Majority 61 (61.6%) newborn babies had received breastfeeding within half an hour. About half of the mothers had any of the feeding problems like feeding less than eight times in 24 h, giving any other food or drinks or is low weight for age. Significantly more mothers with feeding problems had problems in positioning and attachment of infant to the breast as compared with those mothers who did not have any feeding problems.

**Conclusions:**

In the settings, where practice of institutional delivery is high, the staff of healthcare facility should ensure education of the mothers regarding position and attachment of infant to the breast before discharge from the healthcare facility. At the village level, Village Health Nutrition Day (VHND) can be utilized for health education of future mothers and support for the breastfeeding mothers. The IMNCI assessment form for young infant should also include assessment of positioning of infant.

## Introduction

According to the recently developed child growth standards of World Health Organization (WHO) standards, 39% of the children below six months of age are underweight. (1) This finding indicated feeding problems in children below six months of age. In 2003, the WHO recommended infants be fed exclusively on breast milk until six months of age. (2) Breastfeeding provides significant health benefits for infants and mothers. It has also been found to protect against delays in young children’s language and motor skill development. (3) Thus, there is a need for promotion and protection of optimal infant feeding practices for improving nutritional status of children.

In India, the practice of breastfeeding is almost universal, but initiation of breastfeeding is generally quite late and colostrum is discarded. (4) In 1991, Breastfeeding Promotion Network of India (BPNI) was born to protect, promote and support breastfeeding. (5) Recently, the Government of India has undertaken National Rural Health Mission (NRHM), which intends to implement Integrated Management of Neonatal and Childhood Illnesses (IMNCI) through the existing healthcare delivery system. (6) The IMNCI strategy recommended systematic assessment of breastfeeding and emphasized counseling of the mother on proper positioning and attachment of infant to the breast. (7) Information regarding lactating mothers’ skills for proper positioning and attachment of infant while breastfeeding is lacking. Hence, the present study was undertaken to assess breastfeeding skills and practices among the mothers of below six months children in rural Wardha.

## Materials and Methods

### Study area and study subjects

The present cross-sectional study was undertaken in surrounding 23 villages of Kasturba Rural Health Training Centre (KRHTC), Anji, which is a field practice area of Mahatma Gandhi Institute of Medical Sciences (MGIMS), Sewagram, located in Wardha district. The total population of study area was 31 482 with crude birth rate of 16 per thousand population. The study was undertaken during January–March, 2008. The healthcare delivery system of the district comprised of state owned sub-centers (SC), Primary Health Centers (PHCs) at primary level, Rural Hospital (RH) at secondary level and Civil Hospital and two private medical colleges at tertiary level. In the study area, 251 mothers were expected to have children below six months of age. Considering the feasibility, we decided to cover 125 (50%) mothers. Half the villages were randomly selected and all the mothers in the selected villages formed the sample.

### Data collection

Two Auxiliary Nurse Midwives (ANMs) trained in IMNCI strategy could pay house visits to 99 (79%) out of expected 125 lactating mothers during the study period. Over three months of the study period, three visits were made to cover maximum of the sample. After obtaining the written consent from the mothers, they undertook assessment of breastfeeding using IMNCI assessment form for young infants. The information on the background characteristics of mothers like age of the mother, education, socio-economic status from color of ration card, place of delivery and baby’s date of birth was obtained using pre-designed and pre-tested questionnaire.

The infant was checked for feeding problems such as feeding less than eight times in the last 24 h, giving any other food or drinks and low weight for age. Baby’s weight was taken and plotted on the growth chart provided on the IMNCI booklet to assess low weight for age. If the infant was not fed in the previous hour, the mother was asked to put her infant to the breast. If the infant was fed during the last one hour, then the mother was asked when the infant would feed again and the assessment was planned accordingly. Auxiliary Nurse Midwives observed the breastfeeding process for four minutes and recorded the infant’s positioning and attachment to the breast as per IMNCI guidelines. A infant was said to have poor attachment when there was chin not touching the breast, mouth not wide open, lower lip not turned outward or little areola visible above than below the mouth. The poor positioning of the infant was recognized by signs like infant’s neck was twisted or bent forward, infant’s body was turned away from mother, infant’s body not close to mother or only infants neck and head were supported. After recording the observations, if there was difficulty with positioning or attachment, the mother was helped to position and attach her infant better.

### Statistical analysis

The data were entered and analysed using the Epi_Info (version 6.04d) software package. The continuous data is presented as mean values along with their standard errors (SE). The categorical variables are presented as percentages. Z-test was applied to test the difference between two proportions. *P* value less than 0.05 was considered for the statistical significance.

## Results

Total of 99 lactating mothers were studied. The average age of the mothers were 23.5 years (±2.88 SE). The average years of education were 9.9 years (±.3.1 SE). About (24.2%) of mothers were below poverty line and 14 (14.1%) were members in women’s self-help group (SHG). Most of the deliveries, 94 (94.9%) took place in the healthcare facilities, 43.4% of these were in medical college hospital, 38.4% were in district hospital and 7.1% in Primary Health Center [Table 1]. The average age of the infants studied was 3.6 (±0.17 SE) months.

**Table 1:** Characteristics of the responding mothers

Characteristics of mothers	Frequency (%) N=99
Age of the mothers (mean + SE)	23.5 + 2.88
Age of the infants (mean + SE)	3.6 (+ 0.17)
Education of mothers (mean + SE)	9.9 + 3.1
Membership in self-help group	14 (14.1)
Health Insurance	50 (50.5)
Socio-economic status (*N*=99)
Below poverty line	24 (24.2)
Above poverty line	75 (75.8)
Place of delivery
Medical college	43 (43..4)
Civil hospital	38 (38.4)
Primary health center	7 (7.1)
Home	5 (5.1)

Figures in parenthesis are percentages; SE – Standard error

Out of the 99 lactating mothers studied, majority i.e. 61 (61.6%) newborns had received breastfeeding within half an hour. Twenty two (22.2%) babies had received breastfeeding after one hour. Only 10 (10.1%) of mothers were feeding the babies for less than the required minimal eight times in 24 h. Twenty six (26.3%) mothers were giving other food or drinks to the baby. About half (51) of the children had at least one of the feeding problems like feeding less than eight times in 24 h, giving any other food or drinks or is low weight for age.

Among 51 infants who had feeding problem, 22 (43.1%) had at least one problem in positioning the infant to the breast. Sixteen (31.4%) infant’s neck was twisted or bent forward. Fourteen (27.5%) infants had only head and neck supported instead of the whole body and 13 (25.5%) infants’ body was turned away from the mother while breastfeeding. Only 3 (5.9%) infants’ body was not close to the mother [Table 2].

**Table 2:** Assessment of breastfeeding among the mothers having feeding problems and the mothers with no feeding problems

Assessment of breastfeeding	Infant’s with feeding problem (*N* = 51)	Infants with no feeding problem (*N* = 48)	*P* value
Poor positioning of infants to the breast			
Infant’s body not close to mother	3 (5.9)	1 (2.1)	0.262
Infants neck is twisted or bent forward	16 (31.4)	1 (2.1)	0.001
Infant’s body is turned away from the mother	13 (25.5)	1 (2.1)	0.001
Only the infant’s head and neck were supported	14 (27.5)	4 (8.3)	0.001
At least one problem in positioning	22 (43.1)	4 (8.3)	0.001
Poor attachment of infants to the breast			
Chin not touching the breast	11 (21.6)	2 (4.2)	0.010
Mouth not wide open	9 (17.6)	1 (2.1)	0.025
Lower lip not turned outward	10 (19.6)	1 (2.1)	0.005
Less areola below than above	17 (33.3)	2 (4.2)	0.001
At least one problem in attachment	21 (41.2)	2 (4.2)	0.001

Figures in parenthesis are percentages

As presented in Table 2, among 51 infants that had feeding problems, 21 (41.2%) had at least one problem in attaching the infant to the breast. Eleven (21.6%) infants’ chin was not touching the breast, 9 (17.6%) babies did not have mouth wide open, 10 (19.6%) babies’ lip were not turned outward and 17 (33.3%) had little areola visible above than below. Significantly more mothers with feeding problems had problems in positioning and attachment of infant to the breast as compared with those mothers who did not have feeding problems ( *P* < 0.001).

## Discussion

Effective breastfeeding is a function of proper positioning and attachment of child to the mother’s breast. (7) The effective breastfeeding is crucial for getting all the benefits of breastfeeding such as protection against childhood infections, otitis media and other diseases such as juvenile-onset insulin-dependent diabetes mellitus, respiratory diseases and obesity. (8) Mothers also get benefited as there is reduced risk of premenstrual breast cancer, ovarian cancer and hip fracture. (9) The various barriers such as mothers’ poor awareness about exclusive breastfeeding, fear of inadequacy of breast milk and poor support at family level are known; (10) however, the information on mothers’ skills of positioning and attachment of baby to the breast are not available. The most common reasons cited by women for giving up breastfeeding early has been attributed to ineffective positioning and attachment which are preventable. (11)

Our results indicated that about half of the children had any of the feeding problems like feeding less than eight times in 24 h, was given any other food or drinks or was low weight for age even though most of the deliveries took place in the hospital. Significantly more mothers with feeding problems had problems in positioning and attachment of infant to the breast. This was a missed opportunity to educate and support mothers about correct positioning and attachment of infant to the breast for effective breastfeeding. Hence, the skillbased education of the mothers should be emphasized before discharge from the healthcare facility and during the subsequent follow-up visits. This step of imparting specific knowledge and skills to mothers regarding positioning and attachment of the infant to the breast needs to be strengthened under Baby Friendly Hospital Initiative (BFHI). Nyqvist *et al*. recommended education of staff in specific knowledge and skills, ante-natal information about lactation in the event of preterm birth, preference for mother’s own milk, a family-centered and supportive physical environment and early transfer of infants’ care to parents. (12) Law *et al*. demonstrated that a 4-h workshop in a positioning and attachment intervention, using a ‘hands-off’ approach, can increase midwives’ knowledge of breastfeeding support relevant to the immediate post-natal period. It is applicable to all midwives, and could be a cost-effective way of improving the ability of mothers to begin and continue to breastfeed successfully. (11) In India, the ANMs are also being trained in IMNCI strategy, which involves the training on positioning and attachment. The training shall also be extended to the nursing staff of medical colleges and district hospitals. Wallace and Kosmala-Anderson reported only 54% of the midwives in England were competent in positioning and attachment of baby to breast. (13) We could not find such study in India for the purpose of comparison.

According to IMNCI assessment form for young infant, breastfeeding assessment is done when infant has any difficulty feeding, is feeding less than eight times in 24 h, is taking any other food or drinks and has no indication to refer urgently to hospital. We found that significantly more mothers of babies with feeding problems had problems in attachment to the breast as well as in positioning the infant to the breast. Positioning of infant for breastfeeding is important because poor positioning often results in poor attachment, especially in young infants. Additionally, it is to be noted that, IMNCI assessment form for young infant do not take into consideration the position of infant while assessing the breastfeeding. Hence, IMNCI assessment form for young infant should also consider positioning of infant while assessing breastfeeding.

In the present study, only 26.3% of the infants were given food or drinks other than mother’s milk implying that high proportion (73.7%) was exclusive breastfeeding. According to National Family Health Survey III (2005–06), in rural Maharashtra, 55.1% of the infants were below six months who received exclusive breastfeeding. (14) The better practices related to early initiation, frequency of feeding and exclusive breastfeeding in the study area may be attributed to high proportion of hospital deliveries. It may also be due to the implementation of community-based action research focused on health education of the pregnant mothers through celebration of village level monthly *Bal Suraksha Diwas* (BSD, child health day). (10) In BSD, health education was ensured by the ANM, village health worker, and members of Village Health Committee, which is generally missing in field implementation of government health program. Similar health education efforts may be undertaken at village level through monthly Village Health, Nutrition Day (VHND) under National Rural Health Mission. The VHND is seen as a platform for interface between the community and the health system. (15) Britton *et al*. reported that additional professional support was effective in prolonging the breastfeeding, but its effects on exclusive breastfeeding were less clear. However, additional lay support was effective in prolonging exclusive breastfeeding. Thus, the combined support offered by professionals and lay people together improved the breastfeeding – the exclusive breastfeeding and the duration of breastfeeding. (2)

To summarize, in the settings, where practice of institutional delivery is high, the staff of healthcare facility should ensure education of the mothers regarding positioning and attachment of infant to the breast before discharge from the healthcare facility. At the village level, Village Health, Nutrition Day can be utilized for health education of future mothers and support for breastfeeding mothers. The IMNCI assessment form for young infant should also include assessment of positioning of infant to the breast.

## References

[1] Deshmukh PR, Dongre AR, Gupta SS, Garg BS (2007). Implications of newly developed growth standards. Indian J Pediatr.

[2] Britton C, McCormick FM, Renfrew MJ, Wade A, King SE (2007). Support for breastfeeding mothers. Cochrane Database Syst Rev.

[3] Dee DL, Li R, Lee LC, Grummer-Strawn LM (2007). Association between breastfeeding practices and young children’s language and motor skill development. Pediatrics.

[4] Khan ME (1990). Breastfeeding and weaning practices in India. Asia Pac Population J.

[5] Gupta A (2002). 10 years of its work. J Indian Med Assoc.

[6] (2006). Ministry of Health and Family Welfare (MoHFW). National Rural Health Mission (2005-2012), Mission document.

[7] (2005). Government of India, Ministry of Health and Family Welfare. Integrated management of neonatal and childhood illness: Training modules for medical officers.

[8] Advantages of breastfeeding. http://www.breastfeeding.com/all_about/all_about_more2.html.

[9] Benefits of breastfeeding for the mother. http://ayushveda.com/blogs/pregnancy/benefits-of-breastfeeding-forthe-mother/.

[10] Dongre AR, Garg BS (2008). behavior change communication strategy for exclusive breast feeding initiatives: An experience from rural Wardha. Solution exchange for MCH Community Newsletter, Breastfeeding Month Special.

[11] Law SM, Dunn OM, Wallace LM, Inch SA (2007). Breastfeeding Best Start study: Training midwives in hands off positioning and attachment intervention. Mat Child Nutr.

[12] Nyqvist KH, Kylberg E (2008). Application of baby friendly hospital initiative to neonatal care suggestions by Swedish mothers of very preterm infants. J Hum Lact.

[13] Wallace LM, Kosmala-Anderson J (2007). Training needs survey of midwives, health visitors and voluntary-sector breastfeeding support staff in England. Mat Child Nutr.

[14] National Family Health Survey- III. 2005-06. http://www.nfhsindia.org/pdf/MH.pdf.

[15] (2007). Monthly village health nutrition day: Guidelines for AWWs/ ANMs/ASHAs/PRIs. http://www.mohfw.nic.in/NRHM/Documents/VHND_Guidelines.pdf.

